# Reduction of the contaminant fraction of DNA obtained from an ancient giant panda bone

**DOI:** 10.1186/s13104-017-3061-3

**Published:** 2017-12-20

**Authors:** Nikolas Basler, Georgios Xenikoudakis, Michael V. Westbury, Lingfeng Song, Guilian Sheng, Axel Barlow

**Affiliations:** 1 Faculty of Mathematics and Natural Sciences, Institute for Biochemistry and Biology, University of Potsdam, Karl-Liebknecht-Strasse 24-25, 14476 Potsdam, Germany; 2 State Key Laboratory of Biogeology and Environmental Geology, China University of Geosciences, Wuhan, 430074 China

**Keywords:** Ancient DNA (aDNA), Bleach, Pre-digestion, Endogenous content, Palaeogenomics, Paleogenomics, Next generation sequencing (NGS), Giant panda, *Ailuropoda melanoleuca*

## Abstract

**Objective:**

A key challenge in ancient DNA research is massive microbial DNA contamination from the deposition site which accumulates *post mortem* in the study organism’s remains. Two simple and cost-effective methods to enrich the relative endogenous fraction of DNA in ancient samples involve treatment of sample powder with either bleach or Proteinase K pre-digestion prior to DNA extraction. Both approaches have yielded promising but varying results in other studies. Here, we contribute data on the performance of these methods using a comprehensive and systematic series of experiments applied to a single ancient bone fragment from a giant panda (*Ailuropoda melanoleuca*).

**Results:**

Bleach and pre-digestion treatments increased the endogenous DNA content up to ninefold. However, the absolute amount of DNA retrieved was dramatically reduced by all treatments. We also observed reduced DNA damage patterns in pre-treated libraries compared to untreated ones, resulting in longer mean fragment lengths and reduced thymine over-representation at fragment ends. Guanine–cytosine (GC) contents of both mapped and total reads are consistent between treatments and conform to general expectations, indicating no obvious biasing effect of the applied methods. Our results therefore confirm the value of bleach and pre-digestion as tools in palaeogenomic studies, providing sufficient material is available.

**Electronic supplementary material:**

The online version of this article (10.1186/s13104-017-3061-3) contains supplementary material, which is available to authorized users.

## Introduction

Ancient DNA (aDNA) research contributes a wide range of applications and prospects to the field of evolutionary biology [1]. As a result of *post mortem* microbial colonisation, the endogenous fraction of DNA in ancient samples typically makes up less than 1% of the total retrieved DNA (e.g. [2]). The financial costs required to sequence such a sample may therefore be prohibitive. Multiple approaches exist to reduce the contaminant fraction of DNA [3, 4]. Particularly appealing for their simplicity and cost-effectiveness are the exposure of powder from bones or teeth to bleach (sodium hypochlorite solution) [5], a pre-digestion buffer [6], or a combination of both [7]. However, the precise mechanisms underlying these methods remain uncertain, and their exact effect on a particular sample is difficult to accurately predict [5]. The data presented here contribute to a better understanding of these pre-treatment methods.

In this study, three different concentrations of bleach as well as a pre-digestion buffer were applied to the powder of a single ancient bone fragment from a giant panda (*Ailuropoda melanoleuca*). The effects of these different applications on endogenous DNA content, complexity of DNA sequencing libraries, and on characteristic aDNA damage patterns were evaluated.

## Main text

### Ancient bone sample

The investigated bone fragment was found in the sinkhole of Xiaoshuijing, Jiangdong Hill, Tengchong County, Yunnan, China. Its age is 8470 ± 45 years based on radio-carbon dating [8].

Established procedures to avoid modern contamination were followed during DNA extraction and library preparation [9]. Appropriate blank controls were included during all procedures, and consisted of: extraction buffer without bone powder for extraction; nuclease free water instead of DNA extract for library preparation; and Tris–EDTA–Tween (TET) buffer containing 10 mM Tris–HCl (Thermo Fisher 15568-025), 1 mM EDTA (VWR E177-500MLDB) and 0.05% (v/v) Tween-20 (A. Hartenstein CT20), instead of DNA template for all PCRs. No investigator blinding or treatment randomisation was carried out.

### Laboratory procedures

Using a mixer mill (Retsch MM 400), the 1.55 g bone fragment was ground into homogenous powder and then divided into 61 portions of ~ 25 mg and stored at − 20 °C. Eleven portions of 25.1 ± 0.6 mg were used in this study.

Two replicates were carried out for each pre-treatment method. Bleach pre-treatments comprised v/v-dilutions of 0.1, 0.5 and 1.0% bleach (laboratory grade sodium hypochlorite, Sigma Aldrich 425044, 10–15% available chlorine), resulting in ~ 0.015, ~ 0.075 and ~ 0.150% available chlorine, respectively. Following Korlević et al. [5], 1 mL of bleach solution was added to the bone powder, and rotated for 15 min at room temperature. The sample was then pelleted by centrifugation at 16,300×*g*, and the resulting supernatant discarded. Three wash steps were then carried out involving rotation for 3 min in 1 mL water, and centrifugation at 16,300×*g*. For pre-digestion treatment, a pre-digestion buffer containing 0.5% (w/v) *N*-lauroylsarcosine (Sigma Aldrich L9150-50G), 0.5 M EDTA (VWR E177-500MLDB) and 0.25 mg/mL Proteinase K (Promega V3021) was applied to bone powder samples as described by Damgaard et al. [6]. The buffer volume was adjusted for the lower amount of bone powder used here, compared with Damgaard et al. [6], by using 312.5 µL per application instead of 5 mL. Samples were incubated at 37 °C (rather than 50 °C as used in [6]), to match final extraction conditions, and for 45 min (rather than 30 min as used in [6]), as recommended for lower incubation temperatures [6]. After incubation, the tubes were centrifuged for 2 min at 16,300×*g*, and the supernatant discarded. All pre-treatments were immediately followed by DNA extraction. In order to gain comparative values, three bone powder portions were additionally processed without any pre-treatment.

DNA extraction was performed according to Dabney et al. [10] with reduced bone powder input mass, and reduced centrifugation speed of the binding apparatus at approximately 450×*g*. The lower centrifugation speed was chosen based on our previous experience that the binding apparatus can break during centrifugation at higher speeds. To examine the influence of a reduced bone powder input, we performed 12 independent extractions on six cave hyena samples using both 25 and 50 mg of bone powder. Results from this comparison (Additional file 1: Figure S1) showed no consistent evidence of a reduction in DNA yield greater than would be expected based on input bone powder amounts (i.e. DNA yield from 25 mg bone powder being half that obtained from 50 mg bone powder). We interpret this result as indication that no obvious negative influence on extraction efficiency is caused by using this reduced input bone powder amount.

Sequencing libraries were generated from each DNA extract following a single-stranded library preparation protocol [11], which included treatment with uracil-DNA glycosylase (New England Biolabs M0279) and endonuclease VIII (New England Biolabs M0299). The Klenow Fragment of DNA polymerase I (Thermo Fisher Scientific EP0051) was used for the fill-in reaction [5]. 2.5 U/μL of Circligase II (Biozym 131406) was used and the ligation reaction carried out overnight. A quantitative PCR (qPCR) experiment was carried out using 0.2% of the unamplified library to estimate relative library complexities (Additional file 2: Table S1), and to determine the optimal number of cycles for subsequent indexing PCR, representing the inflection point of the respective library amplification curves, corrected for reaction volume and template amount. qPCR was performed on a PikoReal 96 Real-Time PCR machine (Thermo Fisher Scientific TCR0096) with 3 replicates for each library, involving an initial 10 min denaturation at 95 °C, followed by 40 cycles of: 15 s at 95 °C, 30 s at 60 °C, and 1 min at 72 °C. The 10 μL qPCR reaction mix contained 1 μL of diluted library and final concentrations of 1 × SYBR Green qPCR Master Mix (Applied Biosystems 4309155) and 0.2 μM of each primer IS7 and IS8 [11]. The indexing PCR was then performed for the appropriate number of cycles, introducing unique 8 bp indices to both 5′ and 3′ adapters. Final concentrations and PCR were as described by Gansauge and Meyer [11], but using 20 μL of template DNA in a total reaction volume of 80 μL.

DNA sequencing was performed on an Illumina NextSeq 500 sequencing platform, using 500/550 Mid Output v2 (150 cycles, Illumina FC-404-2001) and 500/550 High Output v2 (75 cycles, Illumina FC-404-2005) kits, with a custom read-1 [11] and a custom index-2 [12] sequencing primer. Although paired-end data was acquired for some libraries (GP1-01, GP1-02, GP1-03), only their first reads were used in data analysis, effectively unifying all sequence reads to single-end data of 76 bp length.

### Sequence data analysis

Sequencing reads were trimmed using the software cutadapt (version 1.4.2) [13], requiring a minimum 4 bp overlap for adapter trimming. Duplicates were removed from the trimmed reads using Tally [14] (version 14-020), and 1,500,000 reads subsampled in order to estimate the fragment length distribution of the total DNA (both endogenous and contaminant) recovered using each treatment (Additional file 3: Table S2, Additional file 4: Table S3).

For comparison of endogenous DNA, 1,500,000 trimmed reads ≥ 30 bp were randomly subsampled (Additional file 3: Table S2) and mapped to the reference genome assembly of the giant panda [15] using the “aln” algorithm of BWA [16] (version 0.7.8-r455), with default parameters, and converted to bam format using BWA’s “samse” utility. Using SAMtools [17] (version 0.1.19-44428cd), reads mapping with a phred quality score below 30 were removed (samtools view). The alignment was sorted by 5′ read position (samtools sort), and duplicate reads were collapsed (samtools rmdup). Thymine over-representation at 5′ ends of endogenous DNA fragments was assessed using mapDamage2.0 [18] (version 2.0.2-8-gaeeeffc-dirty).

For the total DNA, guanine–cytosine (GC) contents were obtained directly from trimmed FASTQ files (Additional file 3: Table S2, Additional file 4: Table S3). For endogenous DNA, mapped reads were converted into the FASTQ format using BEDtools [19] (version v2.25.0) for their GC content to be assessed.

### Endogenous content and total DNA recovery

**Fig. 1:**
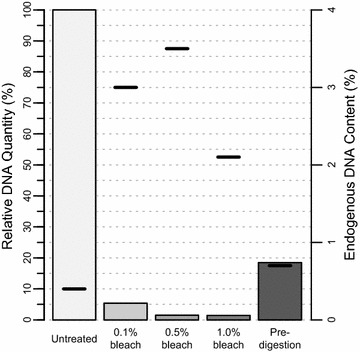
Mean relative DNA quantities and endogenous contents for each pre-treatment. Vertical bars show relative DNA quantities (Additional file 2: Table S1), normalised to the mean of untreated libraries, referring to the left-hand axis. Black horizontal marks show endogenous contents, referring to the right-hand axis

All pre-treated libraries showed higher endogenous contents than untreated ones (Table 1). Considering mean values for each pre-treatment method, the highest increase in endogenous content was observed for 0.5% bleach (ninefold, Fig. 1), which is consistent with the results of previous studies [5]. 0.1 and 1.0% bleach concentrations resulted in eightfold and fivefold mean-increase in endogenous content, respectively. The effect of pre-digestion was less pronounced, providing a twofold increase in endogenous content. Overall DNA retrieval was drastically reduced by all pre-treatment methods, scaling in magnitude with bleach concentration, with the effect of pre-digestion again being less pronounced (Fig. 1). It should be noted that the amounts of retrieved DNA vary within most treatments (by up to 61%, Table 1), which appears to be a common phenomenon in aDNA studies (e.g. [20]).

**Table 1:** Outcome of sequencing experiments

Library	Pre-treatment	Mapped reads (excluding duplicates)	Endogenous content^a^ (%)	Relative DNA quantity^b^ (%)	Mean fragment length	GC content (total/mapped), %
(GP1-01)	None	(4896)	(0.7)	(0.4)	(37.7)	(60/37)
GP1-02	None	2969	0.4	100.0	31.9	64/37
GP1-03	None	3409	0.4	39.0	34.1	63/38
GP1-07	0.1% bleach	28,771	3.5	4.7	36.5	60/37
GP1-10	0.1% bleach	16,898	2.6	2.4	32.8	62/39
GP1-08	0.5% bleach	33,467	3.8	1.3	40.1	57/38
GP1-11	0.5% bleach	23,317	3.1	0.7	36.1	59/39
GP1-09	1.0% bleach	18,865	2.1	1.1	39.6	57/38
GP1-12	1.0% bleach	16,590	2.1	0.7	37.8	59/39
GP1-13	Pre-digestion	5734	0.7	11.7	36.6	62/39
GP1-14	Pre-digestion	4925	0.7	11.3	34.3	62/39

The number of mapped reads, endogenous contents, relative DNA quantities and GC contents of all libraries are reported. GP1-01 (shown in parentheses) is considered an outlier due to an extremely reduced relative DNA quantity in comparison to other untreated libraries (GP1-02 and GP1-03), suggesting failed library preparation, and was not considered for further analysis

^a^Endogenous content was calculated as the quotient of successfully mapped reads (excluding duplicates) and the total number of reads used for mapping

^b^Based on qPCR results (Additional file 2: Table S1). Normalised to GP1-02 (untreated), which yielded the most DNA

The up to ninefold increase of endogenous content in pre-treated libraries implies an equivalent cost reduction for further sequencing attempts of the sample investigated here. However, the large reduction in DNA retrieval rates will be associated with reduced library complexity (i.e. the number of distinct DNA molecules it contains), which may counter any increases in endogenous content by increasing sequence duplication rates. This effect can be mitigated by processing an increased amount of bone powder, provided sufficient material is available.

### Fragment lengths, thymine over-representation and GC content

**Fig. 2:**
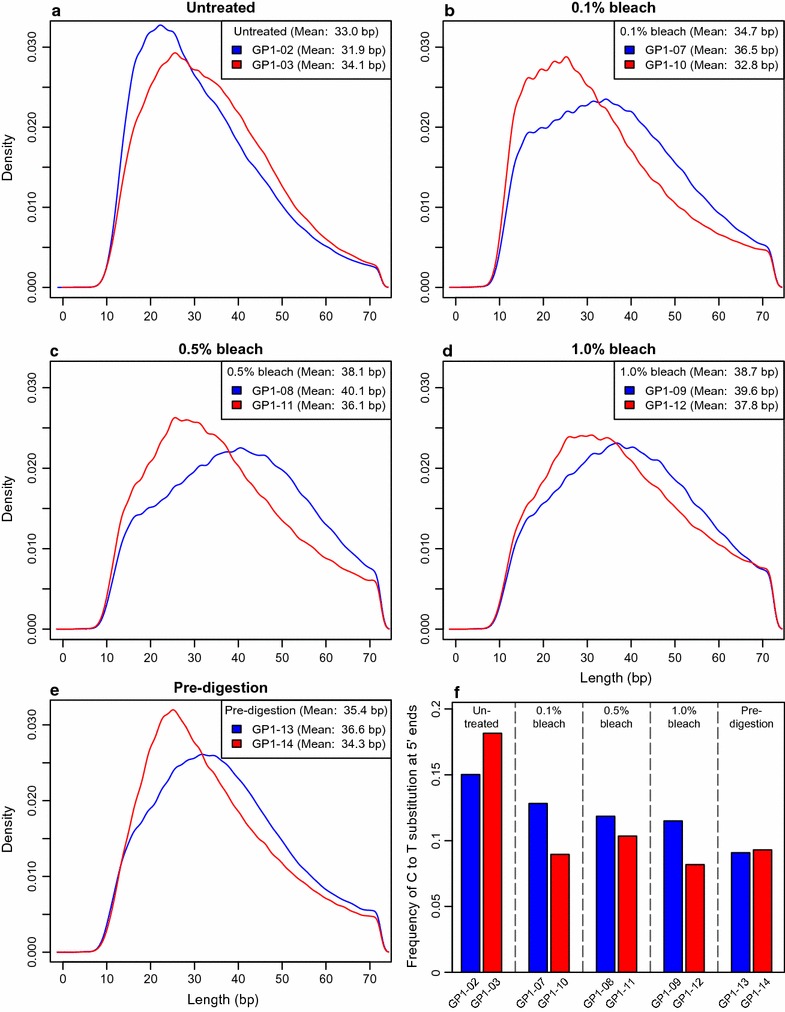
DNA Damage patterns in different pre-treatments. Fragment length distributions (density plots) of total DNA recovered (**a**–**e**, Additional file 4: Table S3). Occurrence of thymine bases at the 5′ ends of endogenous DNA reads where the corresponding base in the reference sequence is a cytosine (**f**)

Mean fragment lengths were generally higher for pre-treated libraries than for untreated ones (Fig. 2a–e). For bleach treated libraries, the length increase appears to be positively correlated with bleach concentration. The mean length of pre-digested libraries was intermediate between that of the 0.1 and 0.5% bleach treated libraries. However, the variation of mean fragment length within replicates was often larger than the difference between treatments. Because of the small number of replicates carried out, any conclusions based on mean values are therefore tentative, and more replicates would be needed in order to robustly test these hypotheses. We also observed consistently reduced levels of thymine overrepresentation in pre-treated libraries compared to untreated ones, albeit with some variation in exact frequencies between replicates (Fig. 2f).

DNA fragmentation and cytosine deamination are typical forms of damage occurring to ancient DNA molecules [21]. The observation that libraries pre-treated with bleach exhibit larger average fragment lengths and reduced cytosine deamination (inferred from thymine over-representation), despite the DNA degrading properties of bleach [22, 23], seems counter-intuitive. We hypothesise that DNA from osteocytes is more protected from both damage and contamination due to its location within the bone’s lacunae [24, 25]. Sample pre-treatment thus enriches for this osteocyte DNA providing both an increase in the endogenous fraction and a reduction in damage rates, but this hypothesis is currently untested.

The GC content of the total recovered DNA was consistently lower in bleached libraries in comparison to untreated ones (Table 1). Smaller reductions in GC content were observed with pre-digestion. For endogenous DNA (mapped reads), GC contents were around 38%, regardless of the pre-treatment method (Table 1), as expected for a mammalian genome [26], indicating no obvious GC content bias introduced by the pre-treatments used in this study.

### Conclusions

Our results add to a growing body of research confirming that bleach and pre-digestion are valuable tools for the study of both palaeogenomes [5–7] and forensics [20, 27]. The majority of published studies have applied these methods to bone and/or tooth samples, however bleach has also been used successfully to remove modern human DNA contamination from hairs (e.g. [28]). To our knowledge, only mitochondrial sequences have been successfully retrieved from ancient hair specimens to date, rendering them potentially less useful than bone samples for studies of ancient nuclear genomes. The wider potential for these pre-treatment methods in the retrieval of genetic data from other ancient or degraded tissues appears largely unexplored, but may represent a beneficial area for future research.

The increases in endogenous content associated with the pre-treatment methods applied here could provide a direct and equivalent reduction in sequencing costs. However, the inevitable reduction of library complexity may counter such gains and necessitate the processing of more sample material. Pre-treatments may be further improved by fine tuning concentrations and incubation times, as well as by comparing their effect on samples from different species, time periods and deposition environments. Even now, samples with very low endogenous DNA contents may become viable for whole-genome sequencing if pre-treatment is applied, greatly increasing the number of potential study subjects.

Finally, the high experimental noise observed in the rates of DNA retrieval (Table 1) and mean fragment lengths (Fig. 2a–e), appear to be common in aDNA research (e.g. [20]). A large number of replicates may therefore be needed for statistical confirmation of the observed trends, particularly when effect sizes are small.

## Limitations

- Only one bone was investigated, precluding broad generalisations as bleach and pre-digestion treatments are known to have sample-specific effects.
- Due to limited amount of material, not enough replicates could be prepared to statistically confirm the results.

## Additional files

**Additional file 1: Figure S1.** DNA mass concentrations of cave hyena extracts. Comparison of DNA yield in 25 mg extractions and 50 mg extractions in cave hyena samples.

**Additional file 2: Table S1.** Results of qPCR experiments. Estimated Number of qPCR cycles to 80 Relative Fluorescence Units for three replicates of each processed library.

**Additional file 3: Table S2.** Terminal commands. One-line UNIX terminal commands used for random subsampling and recording read lengths and GC content.

**Additional file 4: Table S3.** Raw read data. Raw read lengths and GC contents for all libraries.

## Abbreviations

aDNA: ancient DNA
GC content: guanine–cytosine content
NGS: next generation sequencing
qPCR: quantitative PCR
TET: Tris–EDTA–Tween
