# Supplementary figures and images for: Reduction of the contaminant fraction of DNA obtained from an ancient giant panda bone
Source: BMC Res Notes. 2017 Dec 20;10:754. doi: 10.1186/s13104-017-3061-3 (PMC5738828; doi:10.1186/s13104-017-3061-3)

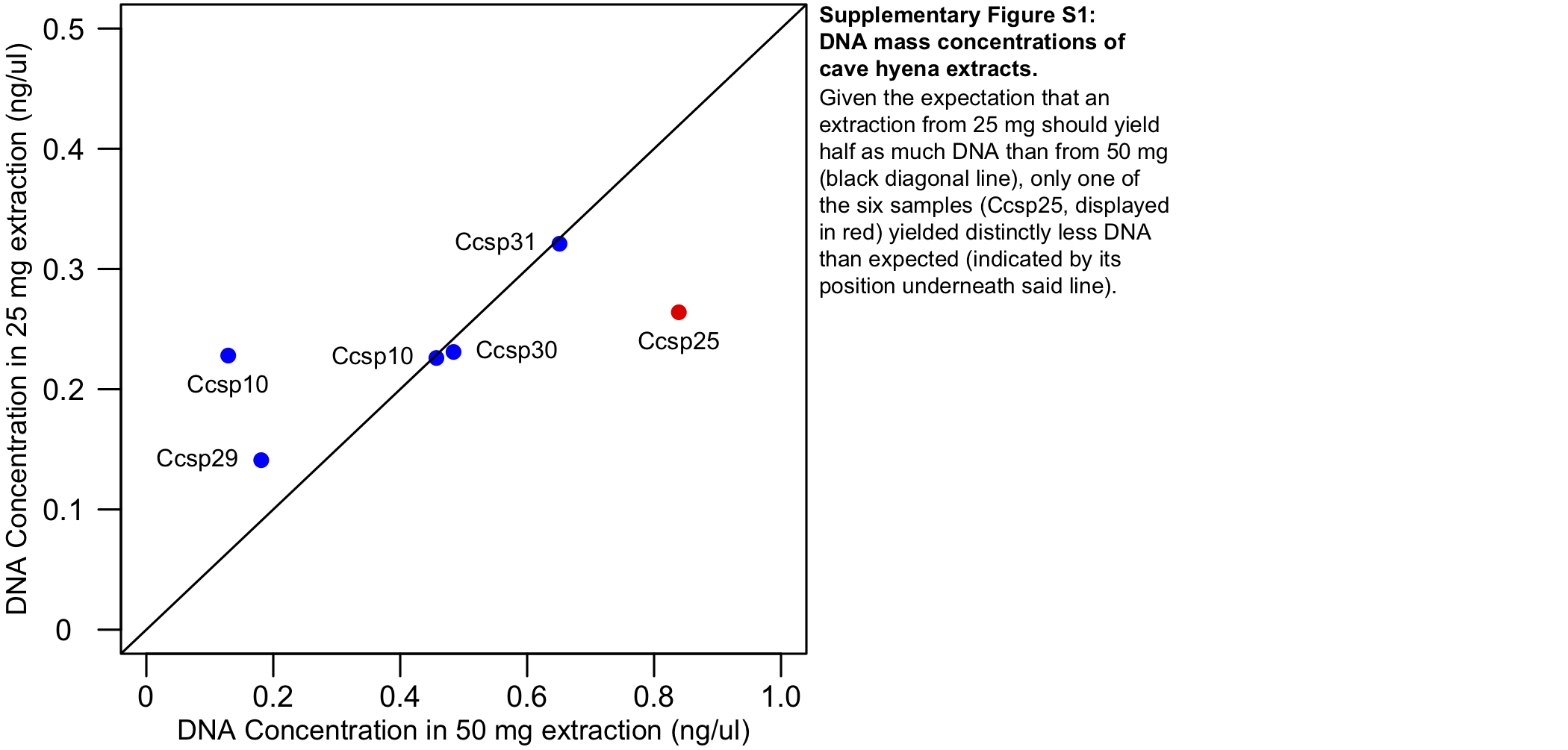

Supplement: Supplementary file 1 — Additional file 1: Figure S1. DNA mass concentrations of cave hyena extracts. Comparison of DNA yield in 25 mg extractions and 50 mg extractions in cave hyena samples. [file 13104_2017_3061_MOESM1_ESM.jpg]
