# Supplementary material for: Reduction of the contaminant fraction of DNA obtained from an ancient giant panda bone
Source: BMC Res Notes. 2017 Dec 20;10:754. doi: 10.1186/s13104-017-3061-3 (PMC5738828; doi:10.1186/s13104-017-3061-3)
Supplement: Supplementary file 2 — Additional file 2: Table S1. Results of qPCR experiments. Estimated Number of qPCR cycles to 80 Relative Fluorescence Units for three replicates of each processed library. [file 13104_2017_3061_MOESM2_ESM.pdf]

Supplementary Table S1: Results of qPCR experiments.

Library	Treatment	Estimated Number of cycles to 80 Relative Fluorescence Units (RFU)			Mean Number of cycles to 80 RFU
		Replicate 1	Replicate 2	Replicate 3	
GP1-01	Untreated	12.12	12.82	12.91	12.62
GP1-02		8.02	8.24	8.22	8.16
GP1-03		9.34	9.58	9.64	9.52
GP1-07	0.1% bleach	12.50	12.64	12.60	12.58
GP1-10		13.50	13.51	13.59	13.53
GP1-08	0.5% bleach	14.64	14.38	14.38	14.47
GP1-11		15.23	15.45	15.34	15.34
GP1-09	1.0% bleach	14.82	14.60	14.69	14.70
GP1-12		15.11	15.41	15.24	15.25
GP1-13	Pre-digestion	11.19	11.32	11.25	11.25
GP1-14		11.13	11.37	11.40	11.30
