# Supplementary material for: Reduction of the contaminant fraction of DNA obtained from an ancient giant panda bone
Source: BMC Res Notes. 2017 Dec 20;10:754. doi: 10.1186/s13104-017-3061-3 (PMC5738828; doi:10.1186/s13104-017-3061-3)
Supplement: Supplementary file 3 — Additional file 3: Table S2. Terminal commands. One-line UNIX terminal commands used for random subsampling and recording read lengths and GC content. [file 13104_2017_3061_MOESM3_ESM.pdf]

Supplementary Table S2: Terminal commands used for random subsampling and recording read lengths and GC content.

<table>
<tr><th>Task</th><th>Code*</th></tr>
<tr><td>Random Sub-sampling</td><td><code>awk '{OFS="\t"; getline seq; getline sep; getline qual; print $0,seq,sep,qual}' INPUT.fastq | shuf -n 1500000 | awk '{OFS="\n"; print $1" "$2,$3,$4,$5}' > SUBSAMPLE.fastq</code></td></tr>
<tr><td>Recording of read lengths and GC content</td><td><code>awk 'NR%4 == 2 && length($0)!=gsub(/N/,"") {print length($0), gsub(/G|C/,"X")/length($0)}' SUBSAMPLE.fastq > LENGTH_AND_GC.txt</code></td></tr>
</table>

* “INPUT.fastq”, “SUBSAMPLE.fastq” and “LENGTH_AND_GC.txt” refer to individual file names.
